# Olive Leaf Mottling Virus: A New Member of the Genus *Olivavirus*

**DOI:** 10.3390/plants13162290

**Published:** 2024-08-17

**Authors:** Ana Belén Ruiz-García, Thierry Candresse, José Malagón, Manuel Ruiz-Torres, Sergio Paz, Ana Pérez-Sierra, Antonio Olmos

**Affiliations:** 1 Instituto Valenciano de Investigaciones Agrarias (IVIA), Ctra CV-315, km 10.7, 46113 Moncada, Valencia, Spain; ana.belen.ruiz@uv.es (A.B.R.-G.); perez_anasie@gva.es (A.P.-S.); 2 UMR BFP, INRAE, University of Bordeaux, CS 20032, 33882 Villenave d’Ornon CEDEX, France; thierry.candresse@inrae.fr; 3 Servicio de Transferencia de Tecnología (STT) de la Generalitat Valenciana, Ctra CV-315, km 10.7, 46113 Moncada, Valencia, Spain; malagon_jos@gva.es (J.M.); paz_ser@gva.es (S.P.); 4 Laboratorio de Producción y Sanidad Vegetal de Jaén, Junta de Andalucía, Sierra Morena, 12b, 23620 Mengíbar, Jaén, Spain; manuelj.ruiz.torres@juntadeandalucia.es

**Keywords:** OLMV, virome, olive, *Olivavirus*, *Closteroviridae*, HTS, detection, identification

## Abstract

Studies of the virome of olive trees with symptoms of leaf mottling by high-throughput sequencing (HTS) revealed the presence of a new virus. Full coding genome sequences of two isolates were determined and consisted of a single RNA segment of 16,516 nt and 16,489, respectively. The genomic organization contained 10 open reading frames (ORFs) from 5′ to 3′: ORF1a, ORF1b (RdRp), ORF2 (p22), ORF3 (p7), ORF4 (HSP70h), ORF5 (HSP90h), ORF6 (CP), ORF7 (p19), ORF8 (p12), ORF9 (p23) and ORF10 (p9). Phylogenetic analyses clustered this virus in the genus *Olivavirus*, family *Closteroviridae,* with the closest species being *Olivavirus flaviolae*, commonly named olive leaf yellowing-associated virus (OLYaV). However, amino acid sequences of all taxonomically relevant proteins showed, in all cases, a divergence higher than 25% between OLYaV and the new virus, indicating that it represents a new species in the genus *Olivavirus* for which the common name of olive leaf mottling virus (OLMV) is proposed. This study represents an advance in the genus *Olivavirus* and provides new insights into the olive virome.

## 1. Introduction

Olive (*Olea europaea*) is an important crop cultivated worldwide. The main production is located in the Mediterranean Basin, with Spain being the largest producer in 2022 according to FAO data [1]. Olive trees can be infected by fungi, bacteria, and viruses that may cause important diseases with a negative impact on production, fruit quality, and tree viability.

Olive leaf yellowing complex (OLYC) is a viral syndrome described three decades ago that regroups at least three different diseases: vein yellowing, leaf yellowing, and yellow mottling and decline [2,3]. The common symptoms of the OLYC diseases are the yellow discoloration of leaves and a low fruit production. In the case of the yellow mottling and decline disease, the symptoms also include necrosis of leaves, extensive defoliation, and dieback [4]. A summary of the viral etiology of the various OLYC diseases and the taxonomical position of the putative causal agents were given by Martelli [4], indicating that olive vein yellowing-associated virus (OVYaV) could be assigned to the genus *Potexvirus*, family *Alphaflexiviridae*, olive leaf yellowing-associated virus (OLYaV) to an undetermined genus of the family *Closteroviridae* and olive yellow mottling and decline-associated virus (OYMDaV) to the genus *Capillovirus* or the genus *Trichovirus*, family *Betaflexiviridae* [4].

In the case of OVYaV, the only knowledge available is that provided by its initial description, where flexuous particles serologically related to the potexviruses papaya mosaic virus (PaMV) and babaco yellow mosaic virus (BaYMV) were observed [2]. However, to date, no genomic information is available for OVYaV. Similarly, very scarce knowledge is available for OYMDaV besides its initial description [3] reporting is discovery and its successful transmission by mechanical inoculation to herbaceous hosts followed by observation of flexuous filamentous particles. As for OVYaV, no genomic information is available for OYMDaV.

A different situation exists for OLYaV, for which the full-length genome coding sequence has recently been determined [5] and several near-complete genomes made available in public databases. This genomic information has recently allowed the virus classification by the International Committee on Taxonomy of Viruses (ICTV) as a new species, *Olivavirus flavioleae*, in the new genus *Olivavirus* of the family *Closteroviridae*.

The olive virome is, however, much more complex and comprises, to date, another 14 viruses that have been described as infecting this crop [6,7,8]. Among them, only strawberry latent ringspot virus has been associated with a disease, in particular to the bumpy fruits syndrome [9]. Hence, olive virology remains a relatively poorly understood field characterized by an absence of comprehensive knowledge on either the genomic features and/or on the biological significance of the various viruses infecting this important crop tree.

The aim of the present work was to gain further knowledge on the olive virome and on the etiology of the OLYC diseases. With this purpose, samples showing olive leaf yellowing and mottling symptoms were analyzed by high-throughput sequencing (HTS). As a result of these efforts, a new virus belonging to the recently recognized genus *Olivavirus* was discovered and is reported here. Future studies are needed to evaluate the biological significance and the epidemiology of this new pathogen.

## 2. Results

### 2.1. Virus-like Symptoms in Olive Trees

**Figure 1:**
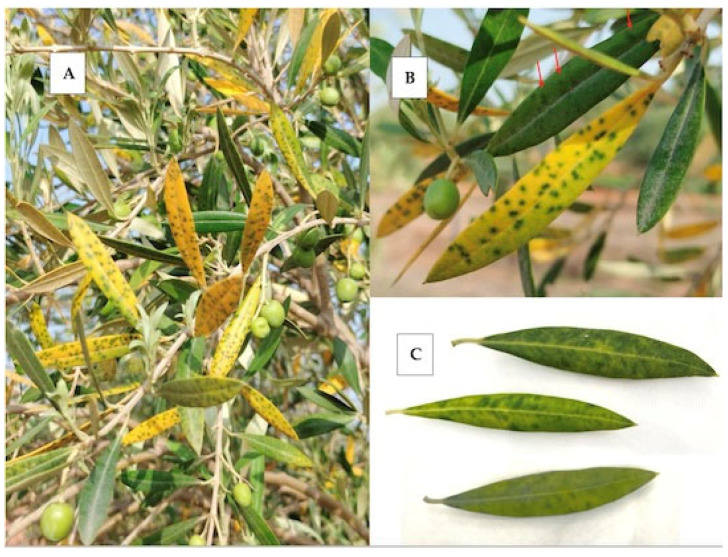
Mottling and yellowing symptoms observed in olive trees. (**A**) Leaf mottling and severe yellow discoloration in sample IVIA158. (**B**) Leaf mottling symptoms in sample IVIA158 in leaves affected or not affected by yellowing. Mottling symptoms are highlighted by arrows in leaves not affected by yellowing. (**C**) Diffuse leaf mottling with limited yellowing symptomatology in sample IVIA167.95.

In July 2023, unusual leaf mottling symptoms, consisting of dark green spots remaining on the otherwise severely yellowed leaf lamina (Figure 1A,B), were observed on olive trees at a germplasm collection of traditional varieties from the Valencian Community located in Moncada, Spain. Dark green spotting was also observed in nonyellowed leaves (Figure 1B). This symptomatology mainly affected one-year-old leaves and was not present on older leaves or on new season leaves. It was accompanied by extensive defoliation. By the end of the year, affected leaves fell down and no mottling or discolored leaves could be observed anymore on affected plants. Neither dieback nor effects on fruit production were observed. Interestingly, in June 2024, the same leaf mottling and yellowing discolorations were observed again on the same trees.

Dark green spotting, more diffuse but this time in the absence of intense yellowing of the leaf lamina (Figure 1C), was also observed among 100 randomly collected samples from a small survey carried out in 2023 by the Plant Health Service in olive orchards located in the area of Jaén, one of the main southern Spain olive growing areas. A total of 11 of the 100 samples were affected by these leaf mottling symptoms but no additional information is available on the status of these plants.

Symptomatic leaves were observed for the presence of mycelium or fungal structures, and attempts at the isolation of fungi were made but neither fungal mycelium nor structures could be observed and no fungal growth resulted from any of the isolation attempts performed.

Two olive plants were selected for HTS analysis based on their different origin, cultivar, and symptomatology: sample IVIA158, cv. Olaya, from Moncada, showing leaf mottling and leaf yellowing; and sample IVIA167.95, cv Picual, from Jaén, showing more diffuse leaf mottling with much less important yellowing symptoms.

### 2.2. Identification of a New Virus by HTS Analysis of Symptomatic Olive Plants

Both sample IVIA158 and sample IVIA167.95 were subjected to an RNASeq HTS analysis with ribodepletion. A total of 42,718,934 reads were obtained for sample IVIA158 after quality trimming. A host genome subtraction step yielded a total of 5,326,835 reads that were used for contigs de novo assembly. Among the 25,554 contigs obtained, a contig of 16,516 nucleotides (nt) (95× average coverage; 10,602 reads) was identified. It had the typical genome organization of *Closteroviridae* members and showed an overall nucleotide identity of 68.2% with the J168.2 isolate of OLYaV (GenBank PP855216). A partial 5′ untranslated region (UTR) of 115 nt (47× average coverage; 86 reads) and a partial 3′ UTR of 227 nt (170× average coverage; 445 reads) could be identified, so the assembled contig seems to represent a near-full-length viral genomic sequence that represents the complete genome coding capacity. This sequence has been deposited in GenBank under the accession number PP869314. The BLAST analysis of the contigs obtained for that sample also revealed the presence of OLYaV. A near-complete genome of OLYaV (isolate IVIA158) could also be reconstructed (GenBank PP855214).

Sample IVIA167.95 yielded 37,522,958 reads after quality trimming, and a total of 8,164,448 reads remained after olive genome subtraction. Contigs de novo assembly yielded 42,939 contigs, among which a contig of 16,489 nt (76× average coverage; 8470 reads) with a similar genomic organization and a nucleotide identity of 68.4% with the Brazilian isolate of OLYaV (MT809205) was identified. A 5′UTR of 115 nt (86× coverage; 165 reads) and a 3′UTR of 204 nt (100×; 219 reads) could be identified, indicating again that a near full-length genome has been recovered. This sequence has been deposited in GenBank under the accession number PP928841. Blast analysis of the contigs from sample 167.95 did not provide evidence for the presence of any other virus.

Sequence comparison of the genomes assembled from the two samples showed that they share 86.8% nucleotide identity and show a similar genome organization, suggesting that these two large contigs could represent two isolates/strains of a new OLYaV-related virus in the family *Closteroviridae*. According to the symptomatology exhibited by the plants where these viral genomes have been identified, we have tentatively named this new virus olive leaf mottling virus (OLMV).

### 2.3. Genome Organization of OLMV

**Figure 2:**
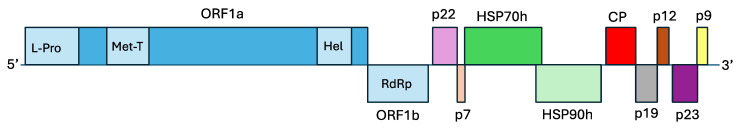
Genome organization of olive leaf mottling virus. L-Pro: papain-like leader protease domain; Met-T: viral methyltransferase domain; Hel: viral helicase domain; RdRp: RNA dependent RNA polymerase; HSP70h: heat shock protein 70 homolog; HSP90h: heat shock protein 90 homolog; CP: coat protein; p22, p7, p19, p12, p23, and p9: hypothetical proteins, numbers indicate the proteins predicted molecular mass in kDa.

A detailed analysis of OLMV genome organization confirmed that it is typical of those shown by *Closteroviridae* members (Figure 2), in agreement with its sequence similarity with OLYaV.

OLMV genome harbors 10 ORFs (Figure 2), including 5 ORFs encoding the five proteins shared by all viruses belonging to the *Closteroviridae* family: ORF1a (with L-Pro leader protease, Met-T methyltransferase and Hel helicase domains), ORF1b (RNA dependent RNA polymerase, RdRp), ORF4 (HSP70h), ORF5 (HSP90h), and ORF6 (CP). A BLASTP analysis [10] of the proteins encoded by these five ORFs against the GenBank database [11] showed highest aa similarities with the corresponding proteins of various isolates of OLYaV, with percentages of identity ranging from 44.96% to 72.20%. Table 1 summarizes the percentages of aa identity for these five proteins for all available complete genomes of OLYaV and OLMV as compared to the OLYaV V64 reference isolate.

In ORF1a, the alignments of the amino acid sequences of the papain-like proteinase domain for other representative closteroviruses allowed the identification of the invariant residues (G, C, P, H, G) [12] at positions 344, 346, 375, 409, and 429, respectively, in both OLMV isolates and the presence of the cleavage site VG/G for the leader protease at positions 429–430, predicting a protease of 49 kDa in both OLMV isolates 158 and 167.95. A conserved domain search using the CDD online tool at the National Center for Biotechnology Information (NCBI) [13] also identified a methyltransferase (pfam01660) domain at positions 645–976 and a helicase domain at positions 2299–2566 in both OLMV 158 and OLMV 167.95. ORF1b, expressed through a +1 ribosomal frameshift typical of closteroviruses [14], encodes a 473 aa long RdRp with a predicted molecular mass of 55 kDa. HSP70h (ORF4), HSP90h (ORF5), and CP (ORF6), are, respectively, 599 aa (67 kDa), 520 aa (61 kDa), and 234 aa (26 kDa). All these proteins have exactly the same length in the two OLMV isolates and showed an aa sequence similarity with OLYaV V64 ranging from 61.46 to 61.97%, 55.43 to 56.40%, and 60.34 to 60.76%, respectively.

Another ORF present in the OLMV genome (ORF2) encodes a thaumatin-like protein of 22 kDa (193 aa). This protein is characteristic of members of the genus *Olivavirus* [5,15,16]. Amino acid sequence similarity for this protein between OLYaV V64 and OLMV isolates ranges between 59.0% and 59.5% (Table 1). The analysis of protein domains with InterPro Scan [17] implemented in Geneious Prime 2023 confirmed the presence of a thaumatin-like domain at position 31–193 aa.

Three more ORFs in the OLMV genome turned out to be related to those of OLYaV: ORF 7, which encodes a protein of 19 kDa with aa similarity levels ranging from 44.8% to 46.8% with the similarly positioned OLYaV p17 hypothetical protein. The isolate OLMV-167.95 produces a protein one aa shorter due an indel affecting the second-to-last 3′ codon so that the p19 is 170 aa long for OLMV 158 and 169 aa long for OLMV 167.95; ORF9, with similarity to the OLYaV p23-encoding ORF (55.0–56.5% aa identity); and ORF10, which encodes a protein of 9 kDa with similarity to OLYaV p10 protein (48.4% to 52.7%) and for which InterProScan predicted by Phobius two potential transmembrane regions at positions 20–43 and 53–74.

ORF3 encodes a 7 kDa protein of 62 aa. BLASTP analysis did not identify any significant similarities with any protein in the GenBank database. Analysis with Geneious Prime 2023 predicted a transmembrane motif at positions 9–29 and with a potential cytoplasmic region (aa 30–62) and a short extracellular region (aa 1–8). InterPro Scan identified in Phobius a similar organization, but with slightly different boundaries for the transmembrane region (aa 6–29). Similar small proteins with predicted transmembrane domains are present in other closteroviruses and in the members of the genus *Olivavirus* [5,15,16].

Lastly, ORF8 encodes a 100 aa long hypothetical protein (p12) with a predicted molecular mass of 12 kDa. This protein of unknown function lacks any BLASTP-detectable identity with proteins in the GenBank database. Similarly to p7, the analysis with Geneious Prime 2023 predicted a transmembrane motif (aa positions 12–32, positions 6–32 for InterPro Scan) and with a potential cytoplasmic region (aa 33–100) and a short extracellular region (aa 1–11).

### 2.4. OLMV Is a New Member of the Genus Olivavirus

The amino acid divergence levels higher than 25% observed between OLMV and OLYaV or any other *Closteroviridae* member in all shared and taxonomically informative proteins (Table 1), together with the presence of a thaumatin-like protein, strongly supports the conclusion that OLMV should be considered as representing a new species in the genus *Olivavirus*.

**Figure 3:**
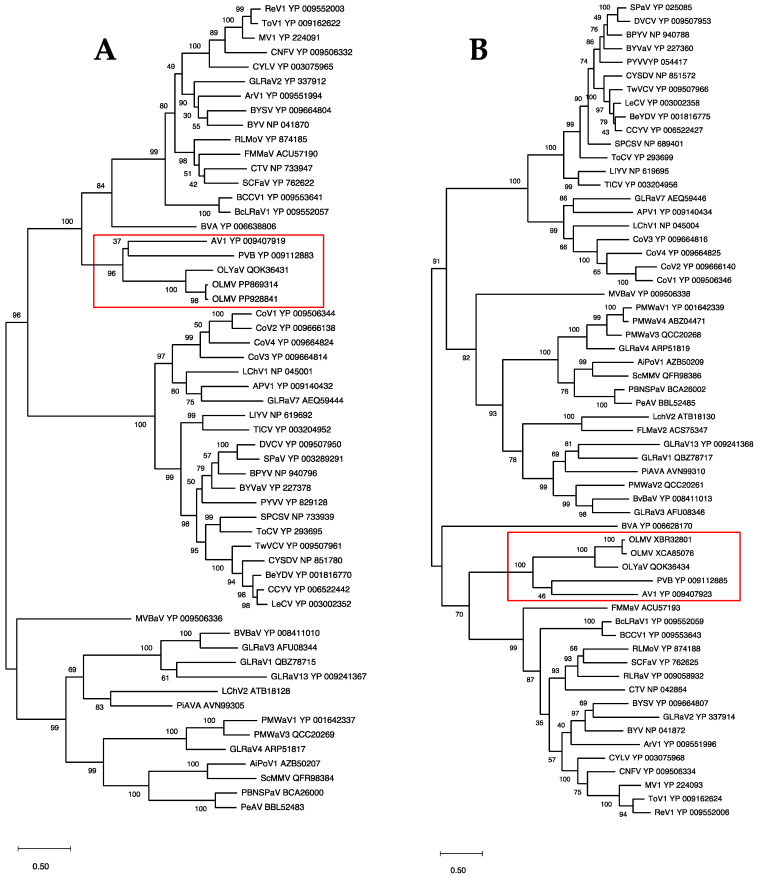
Maximum likelihood phylogenetic trees reconstructed for representative members of family *Closteroviridae* and using multiple alignment of RdRp (**A**) and HSP70h (**B**). Proteins of all isolates are identified by their accession number. The scale bar shows the number of substitutions per site. Bootstrap values (1000 resamplings) are indicated on the branches. The red square highlights the members of the genus *Olivavirus*. Acronyms used are listed in Table S1.

**Figure 4:**
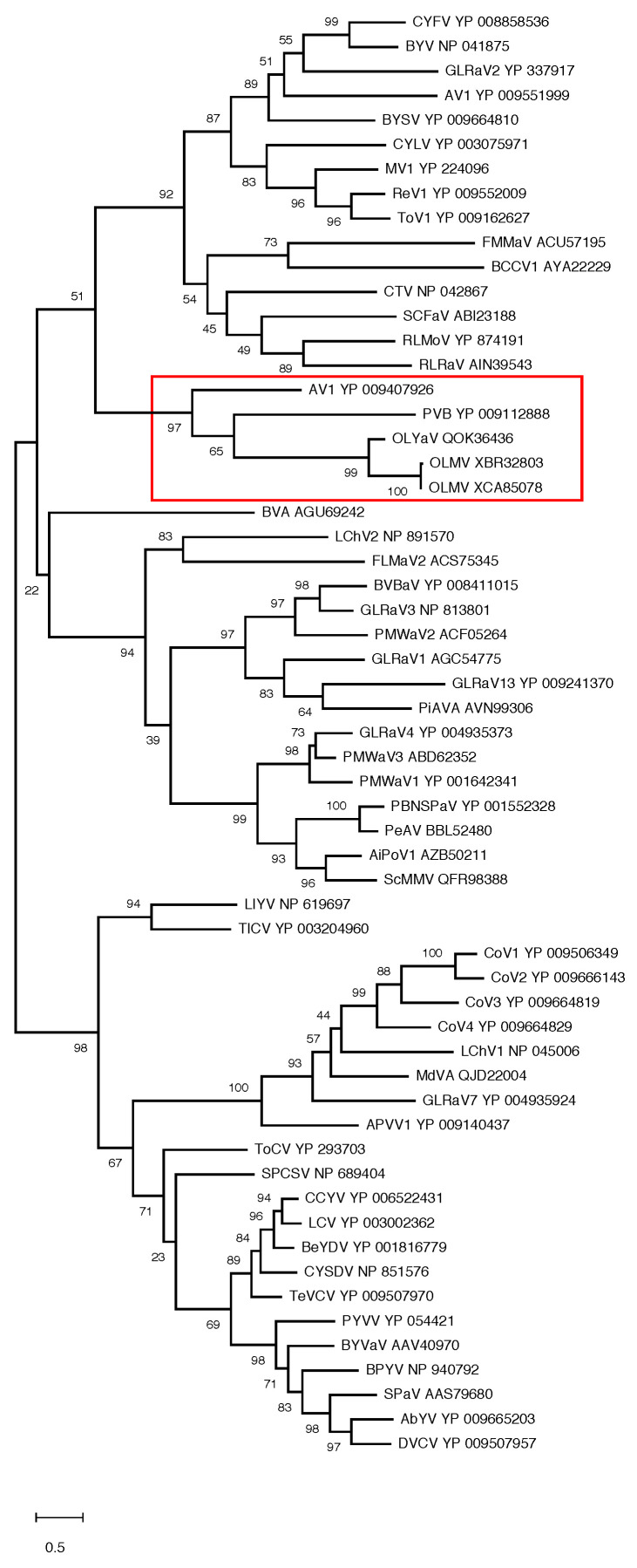
Maximum likelihood phylogenetic trees reconstructed for representative members of family *Closteroviridae* and using multiple alignment of the CP. Proteins of all isolates are identified by their accession number. The scale bar shows the number of substitutions per site. Bootstrap values (1000 resamplings) are indicated on the branches. The red square highlights the members of the genus *Olivavirus*. Acronyms used are listed in Table S1.

In order to confirm the position of OLMV within this genus of the family *Closteroviridae,* phylogenetic analyses were performed using multiple alignments of the RdRp, HSP70h, and CP proteins conventionally used to establish phylogenetic relationships between *Closteroviridae* members. The results show the OLMV group with high bootstrap support together with the Olivaviruses OLYaV, persimmon virus B (PVB), and actinidia virus 1 (AV1) in all three trees (Figure 3 and Figure 4). Similar phylogenetic analyses carried out on ORF1a or HSP90h also clustered OLMV within the genus *Olivavirus*, thus confirming the taxonomic classification of OLMV as a new *Olivavirus*.

### 2.5. RT-PCR Detection of OLMV in Olive Samples

The presence of OLMV in the two original olive samples was confirmed by RT-PCR. For this purpose, specific primers amplifying a fragment of 813 nt and covering the complete CP gene of the virus were designed using the HTS sequences. Both samples, IVIA158 and IVIA167.95, tested positive for OLMV using this method, and Sanger sequencing of amplicons confirmed 100% of the nucleotide sequences obtained by HTS.

In addition, the RT-PCR analysis of the 100 randomly selected olive samples collected in Jaén revealed that OLMV is quite widespread in this area since all 11 symptomatic trees and, in addition, 32 symptomless trees tested positive, resulting in a total of 43 positive trees. It is important to note that no intense leaf yellowing was observed in any of these samples. Sanger sequencing of the amplicons from 20 positive samples (Acc. No PP976337–PP976356) showed that pairwise nt identity ranged from 96.4% to 99.7%, with isolates 167.16 (PP976339) and 167.86 (PP976355) being the most divergent, and isolates 167.29 (PP976344) and 167.56 (PP976348) the most closely related. The analysis of SNPs in the CP gene identified 81 SNPs, 69 of which are silent while 12 of them result in an amino acid (Table 2).

All samples were also tested for the presence of OLYaV. A total of 4 symptomatic as well as 17 symptomless samples were coinfected by both OLYaV and OLMV, while 14 asymptomatic samples were infected by OLYaV alone.

## 3. Discussion

Since the early 1990s, the olive leaf yellowing complex and its associated diseases have remained poorly understood. Similarly, genetic information on viruses present in symptomatic trees and, thus, putatively associated with these diseases remains extremely limited, with the exception of OLYaV, for which complete coding genome sequences have been recently determined [5].

In this study, an unusual leaf mottling combined with severe leaf yellowing symptomatology was observed in an olive orchard in Spain. The subsequent analysis by HTS of a tree showing this symptomatology, as well as that of another tree showing more diffuse leaf mottling in the absence of intense yellow leaf discoloration, has revealed the presence of a new virus, that we here tentatively name olive leaf mottling virus (OLMV). Moreover, two near-complete genomic sequences of OLMV, including all the potential coding sequence, were obtained.

The genomic organization of OLMV involves 10 ORFs, among which are those encoding the five proteins shared by all *Closteroviridae* members, as well as one ORF encoding a thaumatin-like protein typical of Olivaviruses and four ORFs encoding hypothetical proteins. OLMV also shares a high percentage of sequence similarity with OLYaV, although all the proteins shared by these two viruses show more than the 25% of divergence species discrimination cut-off. All these features clearly indicate that OLMV is a new member of the family *Closteroviridae* and belongs to the genus *Olivavirus*.

The presence of OLMV in the two samples analyzed by HTS was confirmed by RT-PCR using specific primers amplifying the full sequence of the CP gene. This PCR-based method was implemented for the detection of OLMV in a small random survey conducted in Jaén, one of the main olive growing areas of Spain, revealing a high incidence of OLMV infection of 43%.

The identification of OLMV in samples showing different leaf mottling symptoms as well as in asymptomatic samples raised the question of the pathogenicity of OLMV. In the trees cv. Olaya from the Valencian community, the mottling consisted of dark green rounded spots in both yellowed and green leaves. Attempts to isolate putative fungi were unsuccessful. In addition, the same trees expressed the same symptomatology in June 2024, a year after the initial symptoms observation, which could be consistent with a viral etiology. In contrast with the yellow mottling and decline disease described a few decades ago by Savino et al. [3], and for which no genomic data are available for the putative agent (OYMDaV), no dieback has been found in any OLMV infected plants. Thus, the leaf mottling observed in the present study likely corresponds to a different syndrome. The mottling observed in the trees from Jaén (cv. Picual) included more diffuse dark green spots and less intense yellow discoloration than observed in the Valencia trees. However, the trees in Jaén were sampled in November, a period of the year in which the trees from the Valencian community had suffered intense defoliation so that only nondiscolored leaves showing green spots were present. This could explain why no intense yellow discoloration was observed in samples from Jaén. In addition, differences related to cultivar, origin, and climatic conditions could also influence symptomatology. Future studies and full monitoring of olive trees affected by OLMV in both single and mixed infection, together with OLYaV in different locations as well as transmission biological tests, will be needed to clarify this issue and, more broadly, the question of OLMV association with symptoms, alone or in mixed infection.

In conclusion, this study reports the discovery of a new virus in the genus *Olivavirus*, family *Closteroviridae*, infecting olive trees that we have tentatively named olive leaf mottling virus (OLMV). This virus could potentially be associated with leaf mottling and extensive defoliation of the trees either alone or in combination with OLYaV, but could also not be involved in the symptoms observed, a situation that clearly deserves further investigation, rendered possible by the development of the specific RT-PCR detection assay reported here. Our results increase the knowledge of the genus *Olivavirus*, provide new information on olive virome, and open new insights into the olive leaf yellowing complex syndrome.

## 4. Materials and Methods

### 4.1. Plant Material

Two olive samples were analyzed by HTS in this study: sample IVIA158 from an olive tree (cv Olaya), showing symptoms of mottling and yellowing in leaves and collected in July 2023 from the Generalitat Valenciana varietal collection grown in Moncada (Spain), and sample IVIA167.95, collected in November 2023 from an olive tree (cv Picual) grown in one of the main producing areas in Jaén (Spain), showing leaf mottling. In addition, 100 asymptomatic olive samples randomly taken from olive trees cultivated in in the Jaén area were collected in November 2023 and analyzed by RT-PCR for the presence of OLMV and OLYaV.

### 4.2. Plan Testing for Fungal Presence

Symptomatic leaves were observed using stereo (Olympus KL 1600 LED, Evident Europe GmbH, Barcelona, Spain) and compound (Olympus BX41, Evident Europe GmbH, Barcelona Spain) microscopes for the presence of mycelium or fungal structures. For fungal isolation, samples were washed under running water and then surface-disinfected with 70% ethanol. Fungal isolations were made on potato dextrose agar (PDA, Scharlab S.L., Valencia, Spain) amended with 0.5% streptomycin sulphate (Thermo Fisher Scientific, Valencia, Spain). Plates were incubated at 20 °C in the dark and checked regularly for signs of fungal growth.

### 4.3. RNA Purification and Preparation of HTS Libraries

Leaves were placed in individual bags (Bioreba, Reinach, Switzerland) with a 1:10 ratio (w:v) of PBS buffer supplemented with 0.2% diethyldithiocarbamate and 2% PVP-10. The Homex 6 homogenizer (Bioreba, Reinach, Switzerland) was then used to grind the plant material. Total RNA was purified from 200 μL of homogenized leaves using the Plant/Fungi total RNA purification kit (Norgen Biotek Corporation, Thorold, ON, Canada) according to the manufacturer’s instructions. Purified RNA was quantified with a DeNovix DS-11 spectrophotometer (DeNovix Inc., Wilmington, DE, USA) and stored at −80 °C until used. HTS libraries were prepared according to Illumina TruSeq Stranded Total RNA Sample Prep Guide (Part #15031048 Rev. E.). Quality control, library construction, and sequencing using the NovaSeq 6000 platform (paired 2 × 150 nt) were performed at Macrogen Inc. (Seoul, Republic of Korea).

### 4.4. Analysis of HTS Datasets and Genome Organization of Olive Leaf Mottling Virus

Raw data were cleaned of Illumina adapters using the Trimmomatic software v.0.39 [18]. Resulting reads were submitted to an olive genome subtraction step using CLC Genomics Workbench v.10.1.1 (Qiagen Bioinformatics, Hilden, Germany) and the GCA 002742605.1 *Olea europaea* genome, the olive mitochondrion genome (MG372121), and the olive chloroplast genome (NC_013707.2). De novo assembled contigs were then obtained using CLC Genomics Workbench with default settings and were annotated by BLAST analysis (BLASTN/X) against local and online virus, viroids, and nt/nr databases. The ORF finder of the Geneious Prime 2023 software was used to predict the proteins encoded by OLMV. Prediction of transmembrane domains and conserved protein domains were performed using Geneious Prime 2023 (Biomatters Ltd., Auckland, New Zealand), InterPro Scan [17], and the conserved domain database [13]. 

### 4.5. Analysis of Encoded Proteins and Phylogenetic Analysis of Olive Leaf Mottling Virus

For the phylogenetic analyses, proteins from representative *Closteroviridae* members were used together with those of OLMV. Multiple alignments of RdRps, HSP70s, and CPs were performed using the MAFFT alignment v.7.490 [19] implemented in Geneious Prime 2023, using default parameters. Phylogenetic trees were reconstructed with the maximum likelihood algorithm implemented in MEGA X [20]. The best substitution model was computed by MEGA X resulting as the Le and Gascuel model [21] implemented with the same equilibrium frequencies (+F) and rates among sites gamma distributed with invariant sites (G+I). In order to obtain representative trees, 1000 bootstraps were used and a representative isolate from each species was included. 

### 4.6. Detection of OLMV and OLYaV by RT-PCR

OLMV-specific primers were designed based on the alignment of the two full-length sequences of OLMV obtained in this study. These primers target the complete CP gene: CP-F (5′ AGA ATG ACT ACA CCA CCA CCT GAG AAC AA 3′) and CP-R (5′ AAG GCA CCC TTC TCC TTG GAA TTT ACG TAG 3′), amplifying a fragment of 813 bp. The RT-PCR reaction was performed using the OneStep PrimeScript RT-PCR kit (Takara Bio Ink., Kasatsu, Japan) following the manufacturer’s instructions. The reaction was performed in a total volume of 20 µL and contained 1 µM of each primer and 100 ng of purified RNA. The amplification protocol included a reverse transcription step at 45 °C for 45 min, a denaturation step at 95 °C for 10 s, and 40 cycles of amplification (95 °C for 15 s, 55 °C for 30 s, and 60 °C for 30 s). Amplicons were purified using the mi-PCR Purification Kit (Metabion International AG, Martinsried, Germany) following the manufacturer’s instructions and were Sanger sequenced. The matrix showing the percentages of nucleotide identity and the identification of SNPs was obtained using Geneious Prime 2023.

OLYaV detection by RT-PCR was performed using specific primers 9527F and 9952R targeting a fragment of 425 bp at the RdRp gene, as reported by Ruiz-García et al. [22].

## Figures and Tables

**Table 1:** Percentages of amino acid identity for the five proteins shared by *Closteroviridae* members and for the thaumatin-like protein specific of Olivaviruses, between OLYaV V64 reference isolate (MW056495) and all OLYaV and OLMV isolates for which complete genome coding sequences are available.

Virus	Isolate	Origin	ORF1a	RdRp (ORF1b)	Thaumatin-like Protein (ORF2)	HSP70h (ORF4)	HSP90h (ORF5)	CP (ORF6)
OLYaV	CS1 (MT809205)	Brazil	94.58%	98.61%	89.47%	88.79%	87.02%	97.86%
	OL2 (OK569886)	Greece	96.26%	98.21%	94.21%	97.79%	96.69%	97.44%
	IVIA158 (PP855214)	Spain	95.79%	98.20%	93.68%	96.94%	95.93%	99.57%
	IVIA160 (PP855215)	Spain	87.80%	96.61%	87.89%	89.64%	87.02%	97.44%
	J168.2 (PP855216)	Spain	95.93%	98.61%	94.74%	97.28%	95.93%	98.29%
	J167.44 (PP855724)	Spain	95.68%	97.41%	93.68%	96.94%	96.71%	96.58%
	J168.4 (PP855725)	Spain	95.71%	97.22%	94.21%	97.11%	97.09%	97.01%
OLMV	OLMV158 (PP869314)	Spain	44.96%	71.99%	58.97%	61.97%	55.43%	60.34%
	OLMV167.95 (PP928841)	Spain	45.73%	72.20%	59.49%	61.46%	56.40%	60.76%

**Table 2:** Analysis of SNPs involved in amino acid modifications in the CP protein.

nt Position ^1^	V1 ^2^/Nº Isolates ^3^	V2 ^4^/Nº Isolates	aa Modification ^5^
31	G/16	A/4	G → S
44	T/12	A/4	F → Y
92	G/19	C/1	S → T
173	C/19	T/1	S → F
337	A/19	G/1	I → V
338	T/17	C/3	I → T
400	A/18	T/2	M → L
402	G/19	A/1	M → I
412	A/19	T/1	T → S
478	T/19	C/1	C → R
496	T/19	G/1	S → A
638	G/15	A/5	R → K

^1^ nt position: nucleotide position in CP gene, numbering according the OLM158 (GenBank PP869314) isolate; ^2^ V1: variant 1 of the SNP; ^3^ Nº isolates: number of isolates sharing this variant; ^4^ V2: variant 2 of the SNP; ^5^ aa modification: change in amino acid between the V1 and V2 variants.

## Data Availability

All data supporting the results and conclusions of the study are contained within the article. Generated sequences have been submitted to GenBank. HTS reads covering the full-length genomes recovered in this study are available upon reasonable request.

## Supplementary Materials

The following supporting information can be downloaded at: https://www.mdpi.com/article/10.3390/plants13162290/s1, Table S1. Acronyms used in all maximum likelihood phylogenetic trees.
