# Supplementary material for: Olive Leaf Mottling Virus: A New Member of the Genus Olivavirus
Source: Plants (Basel). 2024 Aug 17;13(16):2290. doi: 10.3390/plants13162290 (PMC11359234; doi:10.3390/plants13162290)
Supplement: Supplementary file 1 [file plants-13-02290-s001.zip › plants-3123100-supplementary.pdf]

Table S1. Acronyms used in all maximum likelihood phylogenetic trees

Acronym	Virus
AiPoV1	air potato virus 1
APV1	areca palm velarivirus 1
ArV1	arracacha virus 1
AV1	actinidia virus 1
BCCV1	blackcurrant-associated closterovirus 1
BcLRaV1	blackcurrant leafroll-associated virus 1
BeYDV	bean yellow disorder virus
BPYV	beet pseudo-yellows virus
BVA	blueberry virus A
BVBaV	blackberry vein banding associated virus
BYSV	beet yellow stunt virus
BYV	beet yellows virus
BYVaV	blackberry yellow vein-associated virus
BYVaV	blackberry yellow vein-associated virus
CCYV	cucurbit chlorotic yellows virus
CNFV	carnation necrotic fleck virus
CoV1	cordyline virus 1
CoV2	cordyline virus 2
CoV3	cordyline virus 3
CoV4	cordyline virus 4
CTV	citrus tristeza virus
CYLV	carrot yellow leaf virus
CYSDV	cucurbit yellow stunting disorder virus
DVCV	diodia vein chlorosis virus
FLMaV2	fig leaf mottle-associated virus 2
FMMaV	fig mild mottle-associated virus
GLRaV1	grapevine leafroll-associated virus 1
GLRaV2	grapevine leafroll-associated virus 2
GLRaV3	grapevine leafroll-associated virus 3
GLRaV4	grapevine leafroll-associated virus 4
GLRaV7	grapevine leafroll-associated virus 7
GLRaV13	grapevine leafroll-associated virus 13
LChV1	little cherry virus 1
LChV2	little cherry virus 2
LeCV	lettuce chlorosis virus
LIYV	lettuce infectious yellows virus
MV1	mint virus 1
MVBaV	mint vein banding-associated virus
OLYaV	olive leaf yellowing-associated virus
PBNSPaV	plum bark necrosis stem pitting-associated virus
PeAV	persimmon ampelovirus
PiAVA	pistachio ampelovirus A
PMWaV1	pineapple mealybug wilt-associated virus 1
PMWaV2	pineapple mealybug wilt-associated virus 2
PMWaV3	pineapple mealybug wilt-associated virus 3
PMWaV4	pineapple mealybug wilt-associated virus 4
PVB	persimmon virus B
PYVV	potato yellow vein virus
PYVV	potato yellow vein virus
ReV1	rehmannia virus 1
RLMoV	raspberry mottle virus
RLRaV	rose leaf rosette-associated virus
SCFaV	strawberry chlorotic fleck associated virus
ScMMV	sugarcane mild mosaic virus
SPaV	strawberry pallidosis-associated virus
SPCSV	sweet potato chlorotic stunt virus
TICV	tomato infectious chlorosis virus
ToCV	tomato chlorosis virus
ToV1	tobacco virus 1
TwVCV	tetterwort vein chlorosis virus
